# The dual use of research ethics committees: why professional self-governance falls short in preserving biosecurity

**DOI:** 10.1186/s12910-018-0295-0

**Published:** 2018-06-05

**Authors:** Sabine Salloch

**Affiliations:** Institute of Ethics and History of Medicine, University Medicine Greifswald, Ellernholzstr. 1-2, 17487 Greifswald, Germany

**Keywords:** Dual use research of concern, Biosecurity, Research ethics committees, Professionalism, Germany

## Abstract

**Background:**

Dual Use Research of Concern (DURC) constitutes a major challenge for research practice and oversight on the local, national and international level. The situation in Germany is shaped by two partly competing suggestions of how to regulate security-related research: The German Ethics Council, as an independent political advisory body, recommended a series of measures, including national legislation on DURC. Competing with that, the German National Academy of Sciences and the German Research Foundation, as two major professional bodies, presented a strategy which draws on the self-control of science and, inter alia, suggests expanding the scope of research ethics committees (RECs) to an evaluation of DURC.

**Main body:**

This situation is taken as an occasion to further discuss the scope and limits of professional self-control with respect to security-related research. The role of RECs as professional bodies of science is particularly analyzed, referring to the theoretical backgrounds of professionalism. Two key sociological features of professionalism – ethical orientation and professional self-control – are discussed with respect to the practice of biomedical science. Both attributes are then analyzed with respect to the assessment of DURC by RECs.

**Conclusion:**

In conclusion, it is stated that issues of biosecurity transcend the boundaries of the scientific community and that a more comprehensive strategy should be implemented encompassing both professional self-control and legal oversight.

## Background

Dealing with Dual Use Research of Concern (DURC) constitutes a major challenge for research practice and oversight on the local, national and international level. Cases of ethically questionable research in biology and biosciences have attracted considerable even before the term DURC came into use. They included:

- The insertion of the mouse IL-4 gene into the mousepox virus, which led to the production of a superstrain of mousepox affecting mice that had been resistant before [1];
- the de novo artificial synthesis of a live polio virus which paralyzed and killed mice [2];
- the reconstruction of the Spanish flu virus, which had killed between 20 and 50 million people in 1918/19 [3, 4]; and
- the enhancement of the transmissibility of the avian influenza A/H5N1 virus on mammals [5, 6].

Decision-making regarding the legitimacy and value of such research, which obviously entails the risk of being misused in bioterrorism or biowarfare, is required by various actors [7]. *Individual scientists* decide which types of research they undertake and which results they submit for publication. *Scientific institutions*, such as universities, regulate potentially dangerous research activities of their members. *Professional societies* have an influence on the development and implementation of ethical codes of conduct and play an important role in the education of their members. *Publishers* have the final decisive power on whether scientific results are promulgated to the scientific community and the wider public. *National governments* make decisions on research funding and legal controls regarding potentially dangerous materials and technologies. *International bodies*, such as the WHO, finally, can build global policies with respect to the threat of bioterrorism and biowarfare. The responsibility for dealing with security-related research is, thus, spread between a variety of individual and collective actors who can exert their influence on the conduct and promulgation of potentially dangerous scientific enterprises.

The complex discussions about the regulation of biomedical research which bears the potential of being misused for terrorism or warfare are closely related to the question regarding which items can be sensibly thought of as having a ‘dual use’ character. Finding appropriate regulative measures to prevent the misuse of science, therefore, depends greatly on what is the subject of such regulations. Different items are listed in this context, such as ‘material, technology or knowledge’ [8] or ‘research, technologies and artefacts.’ [9]. Other authors also frame the misuse of biological and biomedical data as a DURC problem [10]. Whereas ‘immaterial’ subjects, such as knowledge, can often be transmitted easily and informally, more developed structures are necessary to remove technologies and artefacts from their original research context and use them for terrorist or military purposes. Any regulation of DURC must, therefore, assess the various levels of threat which, inter alia, depend on the necessary expertise and equipment [11]. The US National Science Advisory Board for Biosecurity (NSABB) and the U.S. Government define DURC as:

life sciences research that, based on current understanding, can be reasonably anticipated to provide knowledge, information, products, or technologies that could be directly misapplied to pose a significant threat with broad potential consequences to public health and safety, agricultural crops and other plants, animals, the environment, materiel, or national security [12].

This rather broad definition has been internationally adopted [13, 14], forms a main point of reference in the German discussions and will be followed hereafter. Whereas the definition refers to any form of “life sciences research” the article’s main focus will be on biomedical research and not, for example, on agriculture or veterinary medicine.

In more recent publications, the debate on potentially harmful biomedical research is often framed with respect to so-called “Gain-of-function Research” (GOFR). The term GOFR refers to the creation of biological agents with altered properties [15] or – more concretely – to research which increases the transmissibility and/or the virulence of pathogens [16]. In contrast to DURC, a term which is typically used for issues of biosecurity as the deliberate misuse of science for malevolent purposes (see, for example, NSABB definition above), GOFR is discussed with respect to both: biosecurity and biosafety [16, 17]. The scientific and public debates on GOFR were particularly triggered after the US Government had initiated a pause of funding research on dangerous pathogens (such as influenza, SARS, and MERS viruses) in 2014. This governmental decision was mainly driven by a series of *biosafety* mishaps in the sense of human exposure to pathogens or inadvertent contamination of viral samples. Whereas issues of biosafety are linked with a number of highly relevant ethical questions [17, 18] this article’s focus will be on the biosecurity debate, so on the deliberate misuse of scientific data, products or technologies, which is typically discussed in the context of DURC.

The question of how to prevent the misuse of science is often framed with respect to the two alternatives of governmental oversight by the state or state-like organizations versus scientific self-control [19]. State oversight mechanisms can include legal regulations, such as the US *Patriot Act* and the *Bioterrorism Act*, which control the access, transfer and storage of potentially dangerous biological, chemical or radiological materials [20]. On the international level, multilateral treaties, such as the *Geneva Convention* or the *Biological Weapons Convention* (BWC), ban the development, production and stockpiling of biological weapons and promote their destruction from a global perspective [21]. However concrete suggestions for the national level are missing in this multilateral treaty. An obvious drawback of governmental regulation lies in the fact that it might pose a significant threat to academic freedom [22]. The value of academic freedom can be principally depicted as both, an intrinsic as well as an instrumental good. Evans, however, has convincingly shown that referring to scientific freedom as a good for its own sake can fail with respect to DURC when it has to be critically weighed against the public good of preventing mass casualties [23]. Instrumental appeals to scientific freedom are limited as well, for example because scientific principles such as the confirmation of results can often be achieved without full transparency about potentially dangerous aspects of the research [23].

Scientific self-control, on the other hand, comprises such different measure as the review of research by committees, education and training, and the development and promulgation of professional ethics codes and institutional policies addressing DURC-related issues [24]. Whereas professional self-control can prove beneficial regarding freedom of inquiry, an overemphasis of the internal regulation within the scientific community is laden with problems, such as a bias towards avoiding research restrictions.

This paper illuminates the tension between the two options of scientific self-control versus governmental oversight by using the example of the debates in Germany. Similar to other biomedical developments, extensive discussions on how to regulate DURC started only rather recently in Germany. The situation is shaped by two partly competing suggestions: The German Ethics Council, an independent political advisory body to the Bundestag and Federal Government, suggested a series of measures, including a national legislation on DURC [14]. Competing with that, the National Academy of Sciences and the German Research Foundation, two major professional bodies, made a suggestion which draws on the self-control of science and, inter alia, suggests expanding the scope of existing research ethics committees (RECs) to an evaluation of DURC [25]. This ambiguous situation is taken as an occasion to further analyze the scope and limits of professional self-control in dealing with potentially dangerous research. An exemplary focus addresses the role of RECs in assessing DURC. The critical evaluation of the subjects will be conducted from the perspective of professionalism, which, as a sociological concept, is applied to RECs as bodies of professional self-governance which deal with ethical issues.

## Main text

### DURC as an ethical issue

RECs have a particular focus on the ethical legitimacy of biomedical research projects. It is, therefore, in a first step, necessary to clarify how far dealing with DURC constitutes an *ethical* problem when assessing whether RECs can contribute to the prevention of science misuse. Threats which emanate from the misuse of science and scientific results are obviously related to a range of fields that affect researchers and their institutions, the publishing system, professional and political bodies, and society. Clearly pointing out the ethical concerns which are in play is not easy regarding such a complex and multilayered phenomenon as DURC.

A widespread way of framing the ethical debate on DURC consists of a confrontation between the two main values of the freedom of research versus the duty to avoid causing harm [26]. The advancement of science, mirrored in the individual researcher’s right to freely pursue projects which are beneficial to society, is, thus, contrasted with society’s interest in being saved from severe threats, such as bioweapons or a toxic contamination of food chains. This tension arising between freedom and security is sometimes characterized as the ‘dual-use dilemma’ in public reports and scientific contributions [27–29]. The basic conflict between freedom of science and avoiding harm is spelt out more concretely regarding duties such as stopping research, informing public authorities or not publishing results, which must be discussed in detail with respect to the individual researcher’s obligations [30].

Whereas the ‘ethical dilemma framing’ of DURC might be useful in specific contexts, such as education and awareness-raising, it has considerable shortcomings [31]. Firstly, the ‘ethical dilemma framing’ does not mirror the way in which the dual use issue is experienced and responded to by the scientific community. If scientists and institutions do not experience a ‘dilemma’, they are likely to remain ignorant regarding potential issues of concern. As any attempts to DURC regulation and governance finally aim for the prevention of potentially fatal events, the ‘dilemma framing’ does not serve as a useful strategy when it fails to motivate the relevant stakeholders. Secondly, speaking of a ‘dilemma’ may require broader political negotiations of the structures and practices of biotechnology. If dual-use issues are primarily discussed case by case as a series of dilemmas, this might hinder the politics of governing DURC in the long-term, which should instead function anticipatorily and at a higher political level. Describing DURC as an ethical dilemma, therefore, does not always contribute to the building of adequate policies which are successful and accepted by the relevant stakeholders. Finally, from a more theoretical perspective, the problem of DURC deviates from traditional notions of a ‘dilemma’: Problems of DURC regulation usually do not appear in the sense that we have to decide about *either* being fully permissive with respect to research practice and publication *or* being advocates of a complete prohibition of such research. Instead, complex evaluations are necessary which have to carefully consider each individual case of research conduct, also with respect to its legal, ethical and social implications.

The demand to discuss the ethical issues of DURC from a more comprehensive perspective also arises regarding the role of the precautionary principle (PP), which is often taken as a normative point of reference. The PP as a conceptual tool is usually applied to guide decision-making in risk management. A canonical formulation of this principle is, however, missing. A common feature of all attempts to explicate the PP lies in the fact that they advance precaution in some way. However, the formulas differ in their concrete content and relationship to cost-benefit analysis as another tool for the assessment of risk [32]. The PP can be usefully applied regarding DURC, especially concerning aspects of raising awareness and the establishment of routines which ensure that research is not misused for harmful purposes [8]. Furthermore, it has been argued that the PP is particularly suited for the assessment of situations which are characterized by a high degree of uncertainty [32]. In contrast to risk, uncertain situations are those in which the probability of possible outcomes cannot be quantified on the basis of reliable evidence [33]. As most practical situations contain aspects of risk as well as of uncertainty, a combination of the PP with a cost-benefit analysis is a promising approach, also regarding research which carries the potential of being misused. Applying the PP in an isolated and inflexible way, by contrast, runs the danger of promoting conservative or restrictive options to the disadvantage of a nuanced evaluation of all available evidence on chances and challenges.

In summary, the discussions on DURC as an ethical issue mirror considerations which are typical of the assessment of complex situations affecting certain social groups as well as the entire society. Dealing with the ethical aspects of DURC requires caution and thoughtfulness and has to consider the genuine interests of science and security issues appropriately without framing both points as an irresolvable dilemma.

### Science as a profession: ethical orientation and professional self-control

The notion of professionalism serves as a key concept to understand and interpret structures and developments of modern societies. Physicians and the medical system usually serve as paradigm cases for professionalism in the sociological literature [34, 35]. In contrast to a mere occupation or job, professions are characterized by certain features, structures or functions in society which are analyzed extensively by the sociology of the professions as a scientific subdiscipline. Professions can be traced back historically to the guilds, which were the major organizations for skilled work in the Middle Ages. The guilds served for a public good (the permanent and reliable provision of products and services) and, simultaneously, maintained comfortable working conditions and a high income for their members [36]. Professional organizations, replacing the guilds in modern times, are still powerful at regulating highly important social goods, for example, medical services and research. Compared to clinical medicine, there is a scarcity of literature explicitly addressing biomedical science as a profession [37]. However, the sociology of scientific knowledge, as a related field, highlights the character of science as a social activity and describes the construction of biomedical knowledge through functional and critical analyses of the related occupations [38, 39].

Several features are listed typically as distinguishing professions from other kinds of occupations [40]. Among the most prominent of these characteristics are the presence of professional codes of ethics and the exclusive right to professional self-control. Professions are, thus, characterized, inter alia, by their commitment to self-imposed ethical rules and independency from stately institutions with respect to certain aspects of self-jurisdiction. Regarding the first point, there are numerous codices dealing with the professional and ethical duties of researchers which have been issued, promulgated and promoted internationally by professional bodies [41–43]. Even the call for a ‘Hippocratic Oath for Scientists’ has been raised and discussed vividly [44–46]. Codes of professional conduct have been suggested as one measure to minimize biosecurity risks in the context of DURC in the recent debates on security-related research [12, 14]. They are regarded particularly as an appropriate means to achieve the necessary balance between freedom of research and the need to protect high-value goods, such as national security.

Professional self-control – as a second key feature of professionalism – forms part of the professional authority, which again, emanates from the exclusive body of knowledge that members of the profession have at their command [40]. Professionals use their knowledge and competencies to serve their clients’ needs and maintain societal trust. Professional authority is the basis upon which society confers certain rights to the profession for self-control. Depending on the branch and national context, these rights extend, for example, to regulations of the education curriculum, admission into the profession, structure of the advanced training for specialization or duties towards clients and colleagues. The professional authority is, however, not limitless: It is restricted to those spheres in which the professionals have been educated, therefore, to their distinct field of expertise. The scope and limits of self-control are under continual discussion, for example, regarding the professional organizations’ right to advance ethically controversial positions [47, 48]. With respect to the current debates on DURC, professional organizations in different countries have stressed that they regard self-governance of science as the most effective means of managing the risks of misuse [13, 49]. It is also stressed from other sides that regulative measures towards DURC should, in the first instance, be taken by the scientific community before a state intervention becomes necessary [19]. In the remainder of this article RECs as bodies of professional self-control will be examined regarding their potential to assess DURC-suspected research.

### RECs and the regulation of DURC in Germany

Professional self-control with respect to DURC can be realized by establishing committees particularly dedicated to this task in research institutions or by expanding the spectrum of the RECs or Institutional Review Boards already existing with respect to biosecurity issues. The last option, however, raises questions particularly concerning scope, competencies and responsibilities of the respective boards. The RECs are dedicated to ethical standards and, simultaneously, form bodies of professional self-control. In this degree, they realize the two key components of professionalism discussed before. It seems, thus, worthwhile discussing whether the professional community of science in the form of RECs establishes the appropriate setting to decide about security-related research. As national contexts differ considerably regarding the functions and legal status of RECs, in this article, the German situation is taken as an example for discussing the chances and limits of professional self-control concerning DURC.

The RECs, as interdisciplinary committees, assess ethical, legal and medical aspects of research involving human subjects, biomaterial or data. In fulfilling this task, the committees evaluate such different aspects as scientific originality and relevance, ethical duties towards study participants, and specific legal issues, for example, regarding study registration or monitoring. In this way, RECs protect the research participants as well as the investigators, and contribute to the high quality of research as an important societal goal. The RECs in Germany fulfil their function regarding the physicians’ Professional Code, the German Drug Law and the Medicinal Products Act. According to paragraph 15 of the Professional Code, German physicians have to seek the advice of an REC when undertaking human medical research:

Physicians who participate in a research project which invades the mental or physical integrity of a human being, or uses human body material or data which can be traced to a particular individual, must ensure that advice on questions of professional ethics and professional conduct associated with the project is obtained from an Ethics Committee […] before conducting the research [50].

What is provided by the REC in this professional law context is, however, advice. By contrast, German RECs have an authorization function regarding studies which are subject to drug or medicinal products law. These studies must only be undertaken – next to other conditions – when there is a positive vote from the respective REC. German RECs are generally situated at the medical faculties or the respective State Chamber of Physicians. They are composed of at least six members and their substitutes. It is usually required that one member is qualified judge, one member has professional expertise in medical ethics and that three members have a background in clinical medicine. Expertise in experimental design and statistics is also obligatory as well as a balanced composition of the commission regarding gender [51].

This background information is necessary to understand the impact of the current suggestion of the German Research Foundation and the National Academy of Sciences on the regulation of DURC. These two major professional bodies published their Recommendations for Handling Security-Relevant Research [49] in 2014 and, simultaneously, installed a Joint Committee on the same subject which is supposed to support research institutions in the sustainable implementation of the recommendations. The committee is composed of senior researchers from biomedical sciences, social science, law and theology. In their 2014 recommendations, the two organizations claim jointly that research institutions should define ethics rules for handling security-relevant research and that there should be a specialized REC to advise on issues arising from the implementation of such rules [49]. It is suggested in a subsequent document (‘Progress Report’) from October 2016 that extending the responsibilities of ethics committees already existing would be one option for assessing DURC [25]. By now (March 2018) both options – building new and specialized committees or using the already established committees – have been realized by German research institutions. Furthermore, there are research institutions which are still in their planning phase and others which do not plan to initiate an internal DURC assessment. There is only anecdotal evidence regarding the concrete handling of DURC-suspected research within the committees. The German National Academy of Sciences and the German Research Foundation function as very active promotors of the field who initiate networking and provide further support.

This suggestion of an internal regulation of DURC within scientific institutions competes partly with an opinion paper of the German Ethics Council published in May 2014 [14]. The German Ethics Council provides a thorough analysis of empirical, ethical and legal issues related to DURC. It suggests a bundle of measures to prevent the misuse of dangerous knowledge, products or technologies. The level of awareness is supposed to be raised by introducing the respective topics in academic education, training and public discourse. Furthermore, a national biosecurity code of conduct shall be developed including, for example, regulations on the funding, planning and execution of biosecurity-relevant research. Finally – and divergent from the two main professional bodies characterized above – the German Ethics Council pleads for legally binding regulations on DURC, which include a legal definition and the obligatory consultation of a DURC commission which operates on a national level. Several members of the German Ethics Council even suggested the implementation of a Federal authority to approve suspected DURC on an obligatory basis [14]. Thus, the German Ethics Council, as a political advisory body, supports measures of self-regulation and awareness-raising within the scientific community, but, at the same time, does not exclude legal measures operating in parallel. In the remainder of the paper, whether such a ‘double-tracked’ solution emerges as necessary or whether professional self-regulation alone bears enough potential to prevent the misuse of science will be discussed.

### The ‘dual use’ of RECs – a critical evaluation from the perspective of professionalism

The eligibility of RECs for assessing potentially dangerous research can be discussed from a number of perspectives. The legal situation, for example, is far from clear regarding the juridical basis for assessing security-related research or the liability of committee members and the institutional providers. In addition to this, an unfavorable ‘mission creep’ of RECs can be suspected if their spectrum of tasks is widened to DURC-related issues [52]. Albeit these and other points deserve further attention, there is, so far, a scarcity of literature on the suitability of RECs to assess suspect DURC research or to include a public security-related viewpoint in their ‘portfolio’ [26, 53]. David Resnik comes to the conclusion that RECs should not be burdened with the responsibility of conducting dual use reviews [53]. Instead, he suggests separate dual use or institutional biosafety committees. From Resnik’s point of view, the major task of RECs is the protection of human subjects, and too much extraneous responsibility should be avoided. He argues that ‘Broader social or political issues should be resolved through legislation or administrative policy-making, not through IRB review [53].’

In addition to these important arguments, the perspective from professionalism taken in this article sheds a new light on the role of RECs in assessing security-related research. As argued before, RECs embody two major traits associated with professionalism: They represent the ethical orientation within professional conduct and, simultaneously, form important bodies regarding professional self-control. Both aspects, which are used fruitfully in the assessment of ‘usual’ biomedical research, can, however, be challenged with respect to DURC. The ethical orientation of a profession is directed towards its clients as well as towards the entire society. Orientation towards public welfare thus forms a key trait of professionalism. Professional organizations maintain certain privileges against external forces, while simultaneously ensuring a high quality of services. Regulations, such as those included in professional codes of ethics, are addressed predominantly to members of the profession and sanction deviant behavior within the community. This function of professional ethics, however, contrasts with certain aspects of the regulation of DURC, which could now be required from RECs as professional bodies: The aim of providing good services to clients and society is considerably expanded if RECs are supposed to deal with major security threats. Measures to prevent the abuse of research are not exclusively directed towards members of the professional community (which could, however, happen in case of criminal colleagues), but towards outsiders who use research information, technologies or products for their illegal and highly dangerous purposes. The boundaries of professional ethics are, therefore, markedly transgressed if RECs are used to assess and regulate biosecurity-related issues.

A second key component of professionalism, as depicted above, lies in the professionals’ right to independently regulate issues which form part of their field of expertise. The assessment of suspect DURC research, however, can only partly be seen as falling under this scope. The RECs are composed of experienced researchers, including bioscientists, lawyers and ethicists. They, thus, possess a range of competences which are helpful to understand and estimate, for example, biological backgrounds, chances and challenges, and the research practice of certain experiments. Furthermore, they can assess the compliance with legal regulations and can deal with other ‘traditional’ tasks of research ethics, such as the protection of subjects and risk-benefit analyses. What is missing, however, are specific competencies needed to deal with terroristic threats or biowarfare. Scientists may not always be able to classify research appropriately as being a threat to security. There has not been any established connection to national security agencies and the respective political bodies so far, and it remains doubtful whether top secret information regarding national interests or terrorist threats can be easily shared with RECs at scientific institutions. Finally, a conflict of interest may arise between the scientists’ interest in promoting research and their responsibility to prevent harmful misuse of science [19, 26]. Researchers have a genuine professional interest in advancing knowledge and, in addition, form part of a system which puts them under considerable pressure regarding, for example, presenting new results and publishing. Tensions may arise between the legitimate impetus of knowledge acquisition and the orientation towards public welfare as another key trait of professionalism. Deciding whether a suspect DURC study should be carried out transcends the boundaries of the scientific community in many respects and, in the worst case, can have catastrophic consequences for major parts of the population. It is, therefore, particularly important to weigh the expected gain of knowledge carefully and impartially against the possible consequences, even if they appear unlikely.

The internal regulation of DURC within scientific institutions, as it is currently suggested by the German Research Foundation and the National Academy of Sciences, clearly carries certain advantages related to the preservation of academic freedom and progress in science. Both are important values which, in liberal states, must only be restricted for important reasons. The threats, however, emanating from experiments, such as the reconstruction of a lethal virus, or from technologies which render vaccinations ineffective, could lead to catastrophic events. The level of risk in each case of DURC depends on a variety of different factors which must be evaluated carefully. Among these factors are not only the potential for being used as a weapon and the expertise and equipment needed for misuse [11], but also the political situation and the likelihood of terrorist attacks. A comprehensive assessment encompassing scientific and social and political factors cannot be performed by RECs alone, but requires an extensive strategy including measures of professional self-control as well as aspects of state and international oversight.

The major role of scientific self-control then could lie in measures which promote awareness-raising, education, and a culture of responsibility and research integrity. More concretely, research institutions could initiate undergraduate and graduate curricula on biosecurity, the respective training for staff members and further the exchange between scientific and public discourse. Measures like these have been suggested by the German Ethics Council [14] and, in fact, they might be most powerful in preventing the deliberate misuse of research results, technologies or products for harmful purposes. Activities like these can be considered as concrete implementations of ethical orientation which forms a key trait of professionalism. In addition, professional self-control could extend to interdisciplinary committees which offer collegial advice to those researchers who, by themselves, see a “dual use” potential in their research. These intra-professional ways of dealing with biosecurity risks, however, should not be seen as substitutes for legal regulation in high risk fields of research. The argument from scientific freedom, in many cases, has a limited power in light of the pragmatic circumstances of scientific work and its dissemination [23]. It can thus not be brought forward in general to limit DURC governance to scientific self-control which is, for the reasons demonstrated above, as a context not sufficient for dealing with high-risk DURC.

## Conclusions

Unsurprisingly, the German Research Foundation and the National Academy of Sciences, as two major professional bodies of scientists, promote the position that DURC should be regulated within the boundaries of the scientific system. They highlight the argument that, going this way, the freedom of science is widely protected from political restrictions. A slightly closer look, however, at the foundations of professional ethics and self-control reveals that assessment through RECs as professional bodies might not be appropriately suited to evaluate and, finally, prevent the abuse of scientific knowledge, products or technologies for terroristic or warfare activities. Instead both, ethical orientation as well as professional self-control, should be promoted through measures aiming at awareness-raising, education, and the development of a culture of responsibility and research integrity.

The recent discussions on DURC in Germany are only in their infancy. Context-related and balanced strategies must be developed taking into account the international context. The final success of these attempts will depend ultimately on the willingness to open communication and cooperation on the side of the scientists as well as politicians and representatives of the public. It is to be hoped that serious and constructive processes will be initiated and continued and that effective solutions will be installed before the first severe incident happens which could have disastrous consequences for human health and the environment.

## Abbreviations

BWC: Biological Weapons Convention
DURC: Dual Use Research of Concern
GOFR: Gain-of-function Research
NSABB: National Science Advisory Board for Biosecurity
REC: Research ethics committees
